# Association of Maternal Air Pollution Exposure and Infant Lung Function Is Modified by Genetic Propensity to Oxidative Stress

**DOI:** 10.3390/children11080937

**Published:** 2024-07-31

**Authors:** Dwan Vilcins, Wen Ray Lee, Cindy Pham, Sam Tanner, Luke D. Knibbs, David Burgner, Tamara L. Blake, Toby Mansell, Anne-Louise Ponsonby, Peter D. Sly, Barwon Infant Study Investigator Group

**Affiliations:** 1 Child Health Research Centre, The University of Queensland, South Brisbane, QLD 4101, Australia; d.vilcins@uq.edu.au (D.V.); t.blake@uq.edu.au (T.L.B.); 2 Murdoch Children’s Research Institute, Royal Children’s Hospital, Parkville, VIC 3052, Australia; 3 Department of Paediatrics, University of Melbourne, Parkville, VIC 3052, Australia; 4 Florey Institute, The University of Melbourne, Parkville, VIC 3052, Australia; 5 School of Public Health, The University of Sydney, Sydney, NSW 2006, Australia; 6 Public Health Research Analytics and Methods for Evidence, Public Health Unit, Sydney Local Health District, Camperdown, NSW 2050, Australia; 7 Melbourne School of Population and Global Health, University of Melbourne, Parkville, VIC 3052, Australia

**Keywords:** air pollutants, lung function, oxidative stress, environmental health, children’s health, nitrogen dioxide

## Abstract

Background and objective: The association between air pollution and poor respiratory health outcomes is well established. Children are particularly at risk from air pollution, especially during the prenatal period as their organs and systems are still undergoing crucial development. This study investigated maternal exposure to air pollution during pregnancy and oxidative stress (OS), inflammation, and infant lung function at 4 weeks of age. Methods: Data from the Barwon Infant Study were available for 314 infants. The exposure to NO_2_ and PM_2.5_ were estimated. Infant lung function (4 weeks) was measured by multiple-breath washout. Glycoprotein acetyls (GlycA) (36 weeks prenatal), cord blood, and OS biomarkers were measured in maternal urine (28 weeks). A genetic pathway score for OS (gPFSox) was calculated. Linear regression was used and potential modification by the OS genotype was tested. Results: There was no relationship between maternal exposure to air pollution and infant lung function, or with GlycA or OS during pregnancy. We found an association in children with a genetic propensity to OS between NO_2_ and a lower functional residual capacity (FRC) (β = −5.3 mls, 95% CI (−9.3, −1.3), *p* = 0.01) and lung clearance index (LCI) score (β = 0.46 turnovers, (95% CI 0.10, 0.82), *p* = 0.01). Conclusion: High prenatal exposure to ambient NO_2_ is associated with a lower FRC and a higher LCI score in infants with a genetic propensity to oxidative stress. There was no relationship between maternal exposure to air pollution with maternal and cord blood inflammation or OS biomarkers.

## 1. Introduction

The association of air pollution with respiratory health outcomes is now well established in adult populations, but more work is required to assess the risk to children’s health. Air pollution has been associated with increased mortality [1] and increased hospitalisations due to respiratory disease [2] across all stages of life. Children are particularly at risk from air pollution, including during the prenatal period. Maternal exposure to air pollution is associated with adverse child health outcomes, including some adverse respiratory outcomes [3,4,5], congenital heart defects [6], preterm birth [7], and low birthweight [8]. Previous work has shown that maternal exposure to air pollution is associated with infant (4–5 weeks of age) respiratory changes such as poorer lung function [9], lower minute ventilation, and higher exhaled nitric oxide [10]. Previous evidence has consistently demonstrated a strong association between air pollution with poor respiratory health outcomes. However, there are few studies that have investigated the underlying mechanisms of this association, especially in early life [10,11]. Oxidative stress (OS) and inflammation pathways are emerging as key mediators, where exposure to air pollution induces inflammation and/or OS, which then causes damage on a cellular level, leading to poorer lung health [11]. The data exploring these processes as mediators between air pollution and children’s health outcomes are scarce. Previous work has found that higher levels NO_2_ exposure in the prenatal period was associated with an increase in exhaled nitric oxide in infants, which is indicative of airway inflammation [10]. A prospective study of asthmatic children in Mexico City found that an OS biomarker, malondialdehyde, in expired breath condensate was associated with exposure to ozone, PM_2.5_, and the presence of heavy vehicles, suggesting that children exposed to high levels of air pollution have higher OS responses [11]. In a cross-sectional analysis, exposure to air pollution increased circulating biomarkers of OS (in serum; conjugated dienes, lipo-hydroperoxides, malondialdehyde, and protein carbonylation) in school-aged Mexican children (7–12 years) [12]. A case-control study of Hungarian children revealed an increased risk of infection-induced asthma exacerbation for those children who were more heavily exposed to traffic-related air pollution exposure and had polymorphisms in the NFE2L2 gene, which has a role in the regulation of oxidative stress [13].

To address evidence gaps in this field, our study aims to investigate if maternal exposure to air pollution during pregnancy is associated with decreased infant lung function at 4 weeks of age and the extent to which this association is modified by a child’s genetic risk of OS. Further, we seek to understand if air pollution exposure is associated with OS biomarkers in mothers, or an inflammation marker in maternal blood during pregnancy or cord blood at birth. 

## 2. Methods

### 2.1. Cohort Details

The Barwon Infant Study (BIS) is a longitudinal study of Australian children from the region of Geelong, Victoria. This region includes the city of Geelong and the surrounding areas and has primary industry, rural and coastal areas in addition to the metropolitan city zone. The cohort has several major objectives including the BIS respiratory objective, which seeks to investigate factors influencing respiratory health in children. Women were recruited from two major hospitals (Geelong Hospital which is government-funded and St John of God Hospital which is a private hospital) in the Barwon region. These two hospitals capture over 90% of births in the region. Eligible women were invited to participate in the study at a routine antenatal appointment occurring around 15 weeks of gestation. Recruitment occurred between 2010 and 2013, with ongoing 2-year follow-ups. A total of 1064 women who delivered 1074 infants (10 sets of twins) were enrolled into the study. Participants completed comprehensive questionnaires at 28 weeks antenatal, birth, and 1 month of age, which was supplemented with a physical review at each time point. At the 1 month review, participants were invited to undergo multiple-breath washout (MBW) testing. The 1-month review was completed by 982 infants and MBW testing was attempted in 570 infants whose parents consented to the test, who did not have any respiratory illness, and who fell asleep during the test attempt [14]. Infants with two or more technically acceptable tests were included in this analysis (*n =* 314). The sample size of infants with lung function and maternal OS markers was 257. The study was approved by the Barwon Health Human Research Ethics Committee (HREC 10/24) and written informed consent was obtained from the participating families. Further details on the cohort have been published previously [15]. 

### 2.2. Air Pollutants

We estimated maternal prenatal air pollution exposure using two satellite-based, land-use regression models (Sat-LUR) [16] that we developed and validated and are described in previous studies [17]. The Sat-LUR models were developed for the annual mean concentrations of nitrogen dioxide (NO_2_) and particulate matter ≤ 2.5 µm (PM_2.5_). The indicative spatial resolution of the models is up to 100 m in urban areas and up to 500 m in rural and remote areas [18]. NO_2_ is the most spatially heterogeneous component of traffic-related air pollution (TRAP) and exhibits strong near-traffic gradients; it is therefore a frequently used proxy for TRAP mixtures. Both Sat-LUR models were developed with satellite, land use, and other spatial predictors. The NO_2_ model captured between 66% and 81% of the variability in measured annual NO_2_ concentrations, respectively, with an RMSE of 1.4 to 2 ppb over the time span of the cohort. The PM_2.5_ model captured between 52% and 63% of the variability in PM_2.5_, with a Root Mean Squared Error of 1 to 1.2 µg/m^3^. Participant’s residential addresses during pregnancy were geocoded to 6 decimal places. These were matched with the air quality data to give estimates of the PM_2.5_ and NO_2_ for each participant in the prenatal period, at birth, and at 1 month of age. Where participants changed addresses between follow-ups, their estimated exposure at each property was averaged.

### 2.3. Infant Lung Function

Infant lung function was measured by multiple-breath washout (MBW) with sulphur hexafluoride (SF_6_) [19] at 4 weeks of age during natural sleep [14]. MBW gives indices of lung size (functional residual capacity, FRC) and ventilation distribution (lung clearance index, LCI). The LCI provides an early indication of airway obstruction. Tidal breathing was recorded prior to MBW and the ratio of peak tidal expiratory flow over expiratory time (tPTEF/tE) was calculated. A shortening of the tPTEF/tE ratio has been used previously to indicate airway obstruction [20]. Two records were excluded from the analysis on the basis of implausible lung function values (FRC > 6 L; LCI < 1 lung volume turnovers). As previously stated, there were *n* = 314 children with data for FRC and the LCI; however, due to the nature of the test, we had a slightly higher sample size for tPTEF/tE (*n* = 355).

### 2.4. Inflammation

GlycA is a composite NMR metabolomic marker of glycosylated acute-phase proteins [21]. Previous work in this cohort has shown that GlycA is a superior measure of cumulative inflammation at a younger age than the high-sensitivity C reactive protein [22]. GlycA (mmol/L) was measured in maternal (36 weeks) and cord serum (birth) by nuclear magnetic resonance (NMR) (Nightingale Health, Helsinki, Finland). We tested the associations of air pollution with both maternal GlycA during pregnancy and cord blood GlycA in this study.

### 2.5. Oxidative Stress Biomarkers and Gene Pathway Function Score

Maternal urinary samples at 36 weeks of gestation were collected, processed within 24 h, and stored at −80 °C until analysis. Urinary OS biomarkers, 8-hydroxyguanine (8-OHGua), and 8-hydroxy-2′-deoxyguanosine (8-OHdG) were quantified using liquid chromatography–mass spectroscopy (LC-MS/MS) performed by the Australian National Phenome Centre (Perth, WA, Australia). Briefly, chromatographic separation was performed using an ExionLCTM system (SCIEX; Framingham, MA, USA), reversed-phase separation using a Kinetex C8 2.6 μm 2.1 × 150 mm column (Phenomenex; Lane Cove West, NSW, Australia) at 40 °C, and mass spectrometry detection with electrospray ionisation was evaluated with a QTRAP 6500+ system (SCIEX; Framingham, MA, USA). The lower limit of quantification (LOQ) was 1.5 ng/mL for 8-OHGua and 0.9 ng/mL for 8-OHdG (Cayman Chemical; Ann Arbour, MI, USA). Data acquisition was performed using Analyst^®^1.7.1 and analysed using SCIEX OS Analytics 1.7.0 software (SCIEX; Framingham, MA, USA). The inter-assay coefficient of variation was low (<10%) for the quality control measures. Levels of OS biomarkers below the LOQ were imputed as the LOQ divided by the square root of 2 [23]. Prior to analysis, the urinary OS biomarkers measures were pre-processed to correct for (i) the time interval between urine collection, processing, and storage by fitting a linear model and retaining the residuals [24]; (ii) batch effects [25]; and (iii) urine dilution using specific gravity [26]. Given the left-skewed distribution, a base-2 log transformation was applied to all OS biomarkers for subsequent analyses [27].

A genetic pathway score for OS (gPFS^ox^) was previously developed for this cohort [28]. In brief, DNA samples were extracted from cord blood and infant 12-month whole blood, and whole-genome genotyping was performed. Twelve genes (four pro-oxidant and eight antioxidant) in a minimal OS pathway were identified. For each of these genes, single-nucleotide genetic polymorphisms (SNPs) linked to gene activity were identified using the GTEx database [29,30] and the SNP most strongly associated with expression was chosen (non-tissue specific). After assessing the SNPs’ effect on each identified gene (increased or decreased expression), and the impact of each gene on OS (pro- or antioxidant), a cumulative score was calculated for each participant in the BIS. This score reflects the number of pro-oxidant alleles each individual carries for the OS response pathway, and therefore their propensity towards OS (32). The score was divided by 2 to give a score range of 0–12, and participants with a gPFS^ox^ of ≥8 (which represented the top 20%) were classified as having a high genetic risk of OS.

### 2.6. Covariates

Several covariates were considered for inclusion in the adjusted model. A directed acyclic graph (DAG) was created based on the previous literature and expert knowledge (Supplementary Materials, Figure S1). Several of the variables identified had many records with missing data, and multiple imputations were not appropriate given the volume of missing data. Therefore, a minimal set of confounders was included in the models. Postnatal age with lung function was tested as a covariate but was not found to be important in this cohort. Variable selection was predominately decided by the process of the DAG and expert knowledge by the respiratory scientists and physicians on our team; however, a formal stepwise regression was performed to test the variable set (including those variables with missing data) which found AIC and R^2^ were better in our selected covariate set, and plots of the residuals showed a better fit when variables with a high volume of missingness were removed. The final model included maternal pre-pregnancy body mass index (BMI), maternal age, maternal prenatal smoking (any vs. none), child’s sex, and birthweight.

### 2.7. Statistical Analysis

Variables were described using histograms and basic statistical summaries. Relationships between variables were explored through plotting and correlation analysis using the Pearson product–moment correlation coefficient. Linear regression was used to explore the association of maternal air pollution exposure with infant lung function at 4 weeks of age, OS biomarkers during pregnancy, and inflammation biomarkers during pregnancy (mothers) and from cord blood (child). Modification of the relationship between air pollutants and lung function by the presence of an OS genotype in the child was explored through the use of interaction terms and stratified analysis. PM_2.5_ and NO_2_ were considered as continuous variables for the primary analyses and results are presented for an IQR increase in pollutants. All analyses were performed in R version 4.2.1, 2022 (R Core Team, Vienna, Austria).

#### Sensitivity Analyses

We performed several sensitivity analyses. Socioeconomic status (SES) was considered as a potential confounder. It has previously been shown in this cohort that inflammation partially mediates the relationship between socioeconomic disadvantage and emotional and behavioural problems in children. Education is the socioeconomic indicator recommended by the Organisation for Economic Co-operation and Development for reporting and monitoring socioeconomic inequalities as it is reported with reasonable reliability, can be harmonized across cohorts and countries, generally has few missing data, is relatively stable across adulthood, and is less subject to reverse causality than measures such as income [31,32]. In the BIS, maternal education was self-reported in pregnancy in the following categories: <year 10, year 10, year 12, trade, certificate or diploma, bachelor degree, or postgraduate degree. Multiplicative interaction terms were added for air pollution and maternal smoking in FRC models. PM_2.5_ and NO_2_ were considered as tertiles, instead of as continuous exposures, to test for non-linearity. Models testing tertiles compared medium to low exposure, and high to low exposure. The effect of air pollution exposure at birth and 1 month of age on lung function was also tested. Lastly, we investigated models that included a child’s exposure to environmental tobacco smoke at 1 month of age, as direct exposure to smoke in the home can affect a child’s lung function [33].

## 3. Results

### 3.1. Descriptive Results

There were 314 children who had lung function testing conducted at 4 weeks of age and met all the criteria. Of these, 51% were male. Maternal smoking during pregnancy was present for 15.6% of children. The mean maternal exposure to PM_2.5_ was 7.5 µg/m^3^, (min = 4.9, max = 9.9). The mean maternal NO_2_ was 5.58 ppb (min = 1.9, max = 15.4). The mean FRC in mL/kg was 18.5, the mean LCI score was 6.79, and lung turnover volumes and the tPTEF/tE ratio were 0.37. The cohort summary is presented in Table 1 and Supplementary Table S1.

### 3.2. Air Pollution and Infant Lung Function, and Modification by Genetic Propensity to Oxidative Stress

Table 2 demonstrates the association between air pollution and infant lung function, in the whole population, as well as in children with a genetic propensity to oxidative stress. There was no evidence of a relationship between maternal exposure to air pollution during pregnancy and infant lung function at 4 weeks of age. Maternal exposure to PM_2.5_ was not associated with LCI scores (β = −0.03 turnovers, 95% CI (−0.09, 0.03), *p* = 0.34), FRC (b = 1.3 mls, 95% CI (−0.9, 3.5), *p* = 0.23), or the tPTEF/tE ratio (b = 0.003, 95% CI (−0.010, 0.017), *p* = 0.65) at 4 weeks of age in adjusted models. Similarly, maternal exposure to NO_2_ was not associated with LCI scores (β = 0.01 turnovers, 95% CI (−0.04, 0.06), *p* = 0.68), FRC (β = −0.7 mls, 95% CI (−2.5, 1.1), *p* = 0.46) or the tPTEF/tE ratio (β = 0.003, 95% CI (−0.009, 0.015), *p* = 0.63) at 4 weeks of age in adjusted models.

The relationship between maternal air pollution exposure and lung function was explored in the context of a child’s genetic risk for OS. We found evidence of an association between NO_2_ and reductions in lung function for those children with a gPFSox indicating a high risk of OS. For an IQR increase (ppb) of NO_2_, there was a 5.3 mL decrease in FRC at 4 weeks of age (β = −5.3 mls, 95% CI (−9.3, −1.3), *p* = 0.01). An IQR increase in maternal NO_2_ was not associated with LCI scores (β = 0.08 turnovers, 95% CI (−0.04, 0.20), *p* = 0.18). However, there was a potential for a threshold response.

We did not find a relationship between maternal exposure to PM_2.5_ and either FRC (β = −3.4 mls, 95% CI (−8.4, 1.5), *p* = 0.18) or LCI scores (β = 0.03 turnovers, 95% CI (−0.11, 0.17), *p* = 0.67) in children at high risk of OS. This did not change when using exposure in tertiles (see Table S7). Finally, there was no association with either PM_2.5_ (β −0.01, 95% CI (−0.04, 0.02), *p* = 0.49) or NO_2_ (β 0.01, 95% CI (−0.03, 0.03), *p* = 0.99) and the tPTEF/tE ratio at 4 weeks of age in children at a higher risk of OS.

### 3.3. Air Pollution and Inflammation

Table 3 shows the estimated effect of air pollution on GlycA, a marker of chronic inflammation, in mothers and in cord blood. There was no relationship between maternal exposure to PM_2.5_ (β = −0.01 mmol/L, 95% CI (−0.02, 0.01), *p* = 0.50) or NO_2_ (β = 0.003 mmol/L, 95% CI (−0.012, 0.017), *p* = 0.73) and GlycA in maternal blood in fully adjusted models. Further, there was no relationship between maternal exposure to PM_2.5_ (β = 0.01 mmol/L, 95% CI (−0.02, 0.04), *p* = 0.56) or NO_2_ (β = 0.05 mmol/L, 95% CI (−0.017, 0.028), *p* = 0.64) and GlycA in cord blood in fully adjusted models.

### 3.4. Air Pollution and Oxidative Stress

Table 3 demonstrates the association between air pollution and maternal oxidative stress. There was no relationship between maternal exposure to air pollution and biomarkers for OS during pregnancy or in cord blood. There was no association between PM_2.5_ and urinary 8-OHdG (β = 0.08 ng/mL, 95% CI −0.07, 0.21, *p* = 0.33) or NO_2_ (β = 0.01 ng/mL, 95% CI (−0.12, 0.13), *p* = 0.93) in fully adjusted models. The same was true of urinary 8-OHGua, which was not associated with PM_2.5_ (β = 0.02 ng/mL, 95% CI (−0.16, 0.21), *p* = 0.81) nor NO_2_ (β = 0.03 ng/mL, 95% CI (−0.13, 0.19), *p* = 0.69) in adjusted models.

### 3.5. Sensitivity Analysis

Several sensitivity analyses were performed: using a child’s passive smoking exposure, instead of maternal smoking, as a covariate; an interaction term between air pollution and maternal smoking; air pollutants as tertiles; the effect of SES; and a child’s direct exposure to air pollution and lung function. There were no significant associations in any of our sensitivity analyses and model fit was not improved compared with the main models, with the exception of high exposure to NO_2_ and lung function measures. When including NO_2_ in tertiles, the highest tertile of NO_2_ was associated with differences in both FRC (β = −13.1 mls, 95% CI (−26.1, −0.1), *p* = 0.05) and LCI scores (β = 0.46 turnovers, (95% CI 0.10, 0.82), *p* = 0.01) in children with a genetic propensity to oxidative stress (see Table S7 in the Supplementary Materials, difference in effect from children at low risk of OS is *p =* 0.02 and *p* = 0.007, respectively). A sensitivity analysis was performed using an interaction term between a child’s gPFSox and maternal air pollution for each lung function outcome, but none were significant.

## 4. Discussion

Our study found that high levels of exposure to NO_2_ are associated with lower FRC and higher negative LCI scores in infants with a genetic propensity to oxidative stress. There was no relationship between maternal exposure to air pollution and infant lung function in the whole population. We found no overall association between air pollution and either maternal or cord blood inflammation biomarkers. To the best of our knowledge, only two previous studies have explored the association of maternal air pollution exposure with lung function in infants, and neither sought to test the underlying biological pathways of this association. Further, we could not identify any previous studies that explored maternal exposure to air pollution during pregnancy and OS or inflammation biomarkers. Our results are largely in keeping with those of Latzin et al. [10], who did not find an association with MBW parameters, although they did report an association between PM_10_ and tidal breathing flows. We did not have data on PM_10_ to assess the effect of the larger size fraction in our cohort. Decrue et al. [9] reported an association between minute ventilation and maternal exposure to PM_10_ and NO_2_ in the second trimester, especially in preterm infants; however, they did not find a relationship with the tPTEF/tE ratio and either air pollutant. In our study, there were only 15 participants born preterm (<37 weeks gestational age) with lung function data, and univariate analysis did not find an association between air pollution exposure in the antenatal period and lung function at 4 weeks in preterm infants.

The lack of association between air pollution and OS was surprising. It is difficult to compare our findings with previous work cited in the introduction (10–12) as we used different measures of oxidative stress and inflammation. The work by Latzin found a relationship between maternal exposure to NO_2_ and exhaled nitric oxide, which is a specific measure of airway inflammation compared with urinary GlycA as a more systematic marker. The other studies explored a child’s own exposure, making them not easily comparable. Our sample size was modest for testing associations with air pollution, which tend to have small effect estimates [34], which is an issue in post hoc analysis where the cohort is powered for clinical outcomes. The Barwon Infant Study was designed to minimise participant burden while achieving deep phenotyping, but it has previously been recognised that this may come at a cost of statistical power, especially for uncommon outcomes and those with small effect sizes [15]. It has previously been found that circulating biomarkers are less sensitive to air pollution exposure than more localised lung measures. Zhang et al. [35] found an association between various air pollutants and malondialdehyde in expired breath condensate; however, they found little relationship between air pollutants and yhr oxidised low-density lipoprotein or interleukin-6. Future research on this topic should focus on respiratory-specific markers of oxidation, such as malondialdehyde [11,12] or glutathione sulfonamide. Previous work in this cohort found that a high-risk OS genotype combined with higher exposure to phthalates in utero was associated with an elevated risk of adverse neurodevelopmental outcomes when compared with children who had a genotype that did not increase their risk of OS [36]. It is possible that lung function development is protected by the buffering and defence mechanisms occurring in utero, especially the protection offered by the placenta. There is a well-developed body of literature and meta-analysis showing that endocrine disrupts chemicals cross the placenta and affect the development of key systems [37,38]. However, it is less clear how air pollutants influence the development of children, and which systems are vulnerable. This is an area that requires more research. Lastly, we need to consider the level of air pollution exposure in this cohort, and whether the pollution levels are too low to impact lung development. This is unlikely given that the previous studies were also conducted in communities with historically good air quality. We hypothesized that children with a genotype predisposing them to a higher risk of OS would have a stronger negative association between maternal air pollution exposure and infant lung function, which was true for NO_2_. This indicates that examining the impact of air pollution on those with genetic vulnerability such as those with a low genetic antioxidant capacity is informative.

The Barwon Infant Study is a unique and data-rich cohort, that affords our study several methodological strengths. Lung function testing was performed in non-sedated infants and followed international guidelines [20]. Participants were recruited prospectively in the prenatal period from all eligible members of the population, and therefore there in no selection bias in regard to exposure or outcome. Our air pollution model is externally validated and has been used in previous studies [17]. Urinary oxidative stress biomarkers were measured using the ‘gold-standard’ approach of LC-MS/MS and pre-processed to reduce the number of variables required in the model. However, limitations remain. The sample size was modest, as discussed earlier. Not all parents consented to lung function testing, and only 55% of infants who attempted lung function returned acceptable and reproducible results. Our air pollution model was limited to annual averages, which did not allow us to measure short-term temporal changes or trimester-specific effects. We only had a single time point for the OS biomarkers. We did not have OS biomarkers for the infants. Lastly, our sample was not large enough for sex-specific analysis and may be subject to unmeasured confounding.

## 5. Conclusions

Our study finds no association between maternal exposure to PM_2.5_ or NO_2_ in the prenatal period and infant lung function at four weeks of age, with the exception of NO_2_ for children at higher genetic risk of OS. Further, we did not find an association between maternal exposure to air pollution and OS biomarkers in maternal blood or cord blood.

## Figures and Tables

**Table 1:** Summary of Barwon Infant Study children and mothers, who completed lung function testing at 4 weeks of age (*n* = 314).

Variable	
Male n (%)	160 (51.0)
Birthweight, gm (mean (SD))	3521 (492)
Maternal age, years (mean (SD))	31.98 (5.09)
Maternal pre-pregnancy BMI, kg/m^2^ (mean (SD))	25.63 (5.72)
Any maternal smoking n (%)	48 (15.6)
Child exposed to passive smoke (yes/no) n (%)	10 (3.3)
FRC, mL (mean (SD))	87.8 (15.0)
FRC, mL/kg (mean (SD))	18.5 (3.6)
LCI, lung volume turnovers (mean (SD))	6.79 (0.43)
Ratio of peak tidal expiratory flow over expiratory time (mean (SD))	0.37 (0.1)
Maternal NO_2_ (ppb) (mean (SD))	5.6 (2)
Maternal PM_2.5_ (µg/m^3^) (mean (SD))	7.5 (0.7)
Maternal GlycA (mmol/L)	1.6 (0.2)
Cord blood GlycA (mmol/L)	0.7 (0.2)
Maternal 8-hydroxy-2′-deoxyguanosine (8-OHdG) (ng/mL) (mean (SD)) (log)	1.3 (0.8)
Maternal 8-hydroxyguanine (8-OHGua) (ng/mL) (mean (SD)) (log)	2.9 (1.1)
gPFSox (0–12) (mean (SD))	6.8 (0.9)
gPFSox—high risk > 8) *n* (%)	60 (17.5)

**Table 2:** Results of linear regression models of maternal exposure to air pollution (IQR increase) and infant lung function at 4 weeks of age in the Barwon Infant Study.

**Whole cohort**				
		*Estimates*	*CI*	*p*
LCI *n* = 275	PM_2.5_	−0.03	−0.09, 0.03	0.34
	NO_2_	0.01	−0.04, 0.06	0.68
FRC *n* = 275	PM_2.5_	1.3	−0.9, 3.5	0.23
	NO_2_	−0.7	−2.5, 1.1	0.46
tPTEF/tE *n* = 355	PM_2.5_	0.003	−0.010, 0.017	0.65
	NO_2_	0.003	−0.009, 0.015	0.63
**Children at high risk of oxidative stress**				
		*Estimates*	*CI*	*p*
LCI *n* = 48	PM_2.5_	0.03	−0.11, 0.17	0.67
	NO_2_	0.08	−0.04, 0.20	0.18
FRC *n* = 48	PM_2.5_	−3.4	−8.4, 1.5	0.18
	NO_2_ *	−5.3	−9.3, −1.3	0.01
tPTEF/tE *n* = 65	PM_2.5_	−0.01	−0.04, 0.02	0.49
	NO_2_	0.01	−0.03, 0.03	0.99
**Children at low risk of oxidative stress**				
		*Estimates*	*CI*	*p*
LCI *n* = 222	PM_2.5_	−0.05	−0.12, 0.03	0.23
	NO_2_	−0.01	−0.07, 0.06	0.84
FRC *n* = 222	PM_2.5_	1.23	−2.11, 4.57	0.47
	NO_2_ *	0.87	−1.28, 3.02	0.43
tPTEF/tE *n* = 283	PM_2.5_	0.01	−0.01, 0.02	0.24
	NO_2_	0.01	−0.01, 0.02	0.41

Models adjusted for sex, birthweight, maternal age, maternal pre-pregnancy BMI, and maternal smoking; IQR PM_2.5_ = 0.88, IQR NO_2_ = 2.27; LCI = lung clearance index, FRC = functional residual capacity, and tPTEF/tE = ratio of peak tidal expiratory flow over expiratory time. * Difference between estimates for high vs low risk of oxidative stress is *p* = 0.007.

**Table 3:** Results of linear regression models of maternal exposure to air pollution and maternal and child oxidative stress biomarkers and inflammation in the Barwon Infant Study.

	Air Pollutants	*Estimates*	*CI*	*p*
Oxidative stress biomarkers ^#^				
Maternal 8-hydroxy-2′-deoxyguanosine ^^^	PM_2.5_	0.08	−0.07, 0.21	0.33
	NO_2_	0.01	−0.12, 0.13	0.93
Maternal 8-hydroxyguanine ^^^	PM_2.5_	0.02	−0.16, 0.21	0.81
	NO_2_	0.03	−0.13, 0.19	0.69
Inflammation biomarker				
Maternal GlycA ^^^	PM_2.5_	−0.01	−0.02, 0.01	0.50
	NO_2_	0.003	−0.012, 0.017	0.73
Cord blood GlycA *	PM_2.5_	0.01	−0.02, 0.04	0.56
	NO_2_	0.05	−0.017, 0.028	0.64

^#^ Oxidative stress biomarkers were pre-processed for time intervals between urine collection, processing and storage, batch effects, and urine dilution using specific gravity. ^ Models adjusted for maternal age, maternal pre-pregnancy BMI, and maternal smoking. * Models adjusted for sex, birthweight, maternal age, maternal pre-pregnancy BMI, and maternal smoking.

## Data Availability

Restrictions apply to the availability of these data. Data were obtained from the Barwon Infant Study and are available https://www.barwoninfantstudy.org.au/ with the permission of the Barwon Infant Study Steering Committee.

## Supplementary Materials

The following supporting information can be downloaded at: https://www.mdpi.com/article/10.3390/children11080937/s1, Figure S1: Directed acyclic graph depicted proposed relationships exposure, outcome, and covariates. Green shapes and arrows indicate exposure, blue shapes indicate outcome and its antecedents, including potential mediators. White shapes indicate variables which were included as covariates, while grey shapes are variables proposed to have a relationship on the causal network, but which are unobserved in our models. Maternal TRAP refers to maternal exposure to traffic-related air pollution. Table S1: Summary of exposures and outcomes in the Barwon Infant Study. Table S2: Estimates for lung function at 4 weeks of age (FRC) for maternal exposure to PM_2.5_ and the interaction with maternal smoking in the Barwon Infant Study. Table S3: Estimates for lung function at 4 weeks of age (LCI) for maternal exposure to PM_2.5_, maternal smoking, and a child’s exposure to environmental tobacco smoke at 4 weeks of age in the Barwon Infant Study. Table S4: Estimates for lung function at 4 weeks of age (LCI) for maternal exposure to NO_2_, maternal smoking, and a child’s exposure to environmental tobacco smoke at 4 weeks of age in the Barwon Infant Study. Table S5: Effect estimates for lung function at 4 weeks of age (FRC) for a child’s exposure to PM_2.5_ at different time points, including an interaction with the oxidative stress genotype, in the Barwon Infant Study. Table S6: Effect estimates for lung function at 4 weeks of age (FRC) for a child’s exposure to NO_2_ at different time points, including an interaction with the oxidative stress genotype, in the Barwon Infant Study. Table S7: Results of linear regression models of maternal exposure to air pollution and infant lung function at 4 weeks of age in the Barwon Infant Study, sensitivity using air pollution variable in tertiles.

## References

[1] Chen J., Hoek G. (2020). Long-term exposure to PM and all-cause and cause-specific mortality: A systematic review and meta-analysis. Environ. Int..

[2] Slama A., Śliwczyński A., Woźnica J., Zdrolik M., Wiśnicki B., Kubajek J., Turżańska-Wieczorek O., Gozdowski D., Wierzba W., Franek E. (2019). Impact of air pollution on hospital admissions with a focus on respiratory diseases: A time-series multi-city analysis. Environ. Sci. Pollut. Res..

[3] Gascon M., Vrijheid M., Nieuwenhuijsen M.J. (2016). The Built Environment and Child Health: An Overview of Current Evidence. Curr. Environ. Health Rep..

[4] Dick S., Friend A., Dynes K., AlKandari F., Doust E., Cowie H., Ayres J.G., Turner S.W. (2014). A systematic review of associations between environmental exposures and development of asthma in children aged up to 9 years. BMJ Open.

[5] Schraufnagel D.E., Balmes J.R., Cowl C.T., De Matteis S., Jung S.H., Mortimer K., Perez-Padilla R., Rice M.B., Riojas-Rodriguez H., Sood A. (2019). Air Pollution and Noncommunicable Diseases: A Review by the Forum of International Respiratory Societies’ Environmental Committee, Part 2: Air Pollution and Organ Systems. Chest.

[6] Hu C.-Y., Huang K., Fang Y., Yang X.-J., Ding K., Jiang W., Hua X.-G., Huang D.-Y., Jiang Z.-X., Zhang X.-J. (2020). Maternal air pollution exposure and congenital heart defects in offspring: A systematic review and meta-analysis. Chemosphere.

[7] Han Y., Jiang P., Dong T., Ding X., Chen T., Villanger G.D., Aase H., Huang L., Xia Y. (2018). Maternal air pollution exposure and preterm birth in Wuxi, China: Effect modification by maternal age. Ecotoxicol. Environ. Saf..

[8] Dadvand P., Parker J., Bell M.L., Bonzini M., Brauer M., Darrow L.A., Gehring U., Glinianaia S.V., Gouveia N., Ha E.-h. (2013). Maternal Exposure to Particulate Air Pollution and Term Birth Weight: A Multi-Country Evaluation of Effect and Heterogeneity. Environ. Health Perspect..

[9] Decrue F., Gorlanova O., Salem Y., Vienneau D., Hoogh K.d., Gisler A., Usemann J., Korten I., Nahum U., Sinues P. (2022). Increased Impact of Air Pollution on Lung Function in Preterm versus Term Infants: The BILD Study. Am. J. Respir. Crit. Care Med..

[10] Latzin P., Röösli M., Huss A., Kuehni C.E., Frey U. (2009). Air pollution during pregnancy and lung function in newborns: A birth cohort study. Eur. Respir. J..

[11] Romieu I., Barraza-Villarreal A., Escamilla-Nunez C., Almstrand A., Diaz-Sanchez D., Sly P.D., Olin A. (2008). Exhaled breath malondialdehyde as a marker of effect of exposure to air pollution in children with asthma. J. Allergy Clin. Immunol..

[12] Romero-Calderón A.T., Moreno-Macías H., Manrique-Moreno J.D.F., Riojas-Rodríguez H., Torres-Ramos Y.D., Montoya-Estrada A., Hicks-Gómez J.J., Linares-Segovia B., Cárdenas B., Bárcenas C. (2017). Oxidative stress, lung function and exposure to air pollutants in Mexican schoolchildren with and without asthma. Salud Publica Mex..

[13] Ungvari I., Hadadi E., Virag V., Nagy A., Kiss A., Kalmar A., Zsigmond G., Semsei A.F., Falus A., Szalai C. (2012). Relationship between air pollution, NFE2L2 gene polymorphisms and childhood asthma in a Hungarian population. J. Community Genet..

[14] Gustafsson P.M., Bengtsson L., Lindblad A., Robinson P.D. (2017). The effect of inert gas choice on multiple breath washout in healthy infants: Differences in lung function outcomes and breathing pattern. J. Appl. Physiol..

[15] Vuillermin P., Saffery R., Allen K.J., Carlin J.B., Tang M.L., Ranganathan S., Burgner D., Dwyer T., Collier F., Jachno K. (2015). Cohort Profile: The Barwon Infant Study. Int. J. Epidemiol..

[16] Knibbs L.D., Hewson M.G., Bechle M.J., Marshall J.D., Barnett A.G. (2014). A national satellite-based land-use regression model for air pollution exposure assessment in Australia. Environ. Res..

[17] Knibbs L.D., Coorey C.P., Bechle M.J., Cowie C.T., Dirgawati M., Heyworth J.S., Marks G.B., Marshall J.D., Morawska L., Pereira G. (2016). Independent Validation of National Satellite-Based Land-Use Regression Models for Nitrogen Dioxide Using Passive Samplers. Environ. Sci. Technol..

[18] Ahmed S.M., Mishra G.D., Moss K.M., Yang I.A., Lycett K., Knibbs L.D. (2022). Maternal and Childhood Ambient Air Pollution Exposure and Mental Health Symptoms and Psychomotor Development in Children: An Australian Population-Based Longitudinal Study. Environ. Int..

[19] Vukcevic D., Carlin J.B., King L., Hall G.L., Ponsonby A.L., Sly P.D., Vuillermin P., Ranganathan S. (2015). The influence of sighing respirations on infant lung function measured using multiple breath washout gas mixing techniques. Physiol. Rep..

[20] Stocks J., Sly P., Tepper R., Morgan W. (1996). Infant Respiratory Function Testing.

[21] Otvos J.D., Shalaurova I., Wolak-Dinsmore J., Connelly M.A., Mackey R.H., Stein J.H., Tracy R.P. (2015). GlycA: A Composite Nuclear Magnetic Resonance Biomarker of Systemic Inflammation. Clin. Chem..

[22] Collier F., Ellul S., Juonala M., Ponsonby A.-L., Vuillermin P., Saffery R., Burgner D., on behalf of the Barwon Infant Study Investigator Group (2019). Glycoprotein acetyls (GlycA) at 12 months are associated with high-sensitivity C-reactive protein and early life inflammatory immune measures. Pediatr. Res..

[23] Hornung R.W., Reed L.D. (1990). Estimation of Average Concentration in the Presence of Nondetectable Values. Appl. Occup. Environ. Hyg..

[24] Mortamais M., Chevrier C., Philippat C., Petit C., Calafat A.M., Ye X., Silva M.J., Brambilla C., Eijkemans M.J.C., Charles M.-A. (2012). Correcting for the influence of sampling conditions on biomarkers of exposure to phenols and phthalates: A 2-step standardization method based on regression residuals. Environ. Health.

[25] Engel S.M., Villanger G.D., Nethery R.C., Thomsen C., Sakhi A.K., Drover S.S.M., Hoppin J.A., Zeiner P., Knudsen G.P., Reichborn-Kjennerud T. (2018). Prenatal Phthalates, Maternal Thyroid Function, and Risk of Attention-Deficit Hyperactivity Disorder in the Norwegian Mother and Child Cohort. Environ. Health Perspect..

[26] Levine L., Fahy J.P. (1946). Evaluation of urinary lead excretion for persons at work. J. Ind. Hyg. Toxicol..

[27] Pham C., Thomson S., Chin S.-T., Vuillermin P., O’Hely M., Burgner D., Tanner S., Saffery R., Mansell T., Bong S. Maternal oxidative stress during pregnancy associated with emotional and behavioural problems in early childhood: Implications for fetal programming. 2023, accepted in Molecular Psychiatry.

[28] Tanner S., Thomson S., Drummond K., O’Hely M., Symeonides C., Mansell T., Saffery R., Sly P.D., Collier F., Burgner D. (2022). A Pathway-Based Genetic Score for Oxidative Stress: An Indicator of Host Vulnerability to Phthalate-Associated Adverse Neurodevelopment. Antioxidants.

[29] Lonsdale J., Thomas J., Salvatore M., Phillips R., Lo E., Shad S., Hasz R., Walters G., Garcia F., Young N. (2013). The genotype-tissue expression (GTEx) project. Nat. Genet..

[30] GTEx Portal. https://gtexportal.org/home/.

[31] Duncan G.J., Daly M.C., McDonough P., Williams D.R. (2002). Optimal indicators of socioeconomic status for health research. Am. J. Public Health.

[32] Mackenbach J., Menvielle G., Jasilionis D., de Gelder R. (2015). Measuring Educational Inequalities in Mortality Statistics. https://www.oecd-ilibrary.org/economics/measuring-educational-inequalities-in-mortality-statistics_5jrqppx182zs-en.

[33] Carlsen K.-H., Carlsen K.C.L. (2008). Respiratory effects of tobacco smoking on infants and young children. Paediatr. Respir. Rev..

[34] Verbeek J.H., Whaley P., Morgan R.L., Taylor K.W., Rooney A.A., Schwingshackl L., Hoving J.L., Vittal Katikireddi S., Shea B., Mustafa R.A. (2021). An approach to quantifying the potential importance of residual confounding in systematic reviews of observational studies: A GRADE concept paper. Environ. Int..

[35] Zhang X., Staimer N., Gillen D.L., Tjoa T., Schauer J.J., Shafer M.M., Hasheminassab S., Pakbin P., Vaziri N.D., Sioutas C. (2016). Associations of oxidative stress and inflammatory biomarkers with chemically-characterized air pollutant exposures in an elderly cohort. Environ. Res..

[36] Ponsonby A.-L., Symeonides C., Saffery R., Mueller J.F., O’Hely M., Sly P.D., Wardrop N., Pezic A., Mansell T., Collier F. (2020). Prenatal phthalate exposure, oxidative stress-related genetic vulnerability and early life neurodevelopment: A birth cohort study. Neurotoxicology.

[37] Tang Z.-R., Xu X.-L., Deng S.-L., Lian Z.-X., Yu K. (2020). Oestrogenic Endocrine Disruptors in the Placenta and the Fetus. Int. J. Mol. Sci..

[38] Zhao X., Peng S., Xiang Y., Yang Y., Li J., Shan Z., Teng W. (2017). Correlation between Prenatal Exposure to Polybrominated Diphenyl Ethers (PBDEs) and Infant Birth Outcomes: A Meta-Analysis and an Experimental Study. Int. J. Environ. Res. Public Health.

