# Supplementary material for: Association of Maternal Air Pollution Exposure and Infant Lung Function Is Modified by Genetic Propensity to Oxidative Stress
Source: Children (Basel). 2024 Jul 31;11(8):937. doi: 10.3390/children11080937 (PMC11352943; doi:10.3390/children11080937)
Supplement: Supplementary file 1 [file children-11-00937-s001.zip › children-3083615-supplementary.pdf]

Association of Maternal Air Pollution Exposure and Infant Lung Function Is Modified by Genetic Propensity to Oxidative Stress

Dwan Vilcins [1], Wen Ray Lee [1], Cindy Pham [2,3], Sam Tanner [4], Luke D. Knibbs [5,6], Dave Burgner [2,3], Tamara L. Blake [1], Toby Mansell [2,3], Anne-Louise Ponsonby [2,4,7], Peter D. Sly [1,*] and Barwon Infant Study Investigator Group [2]

1 Child Health Research Centre, The University of Queensland, South Brisbane, QLD 4101, Australia; d.vilcins@uq.edu.au (D.V.); t.blake@uq.edu.au (T.L.B.)
2 Murdoch Children's Research Institute, Royal Children's Hospital, Parkville, VIC 3052, Australia
3 Department of Paediatrics, University of Melbourne, Parkville VIC 3052, Australia
4 Florey Institute, The University of Melbourne, Parkville, VIC 3052, Australia
5 School of Public Health, The University of Sydney, Sydney, NSW 2006, Australia
6 Public Health Research Analytics and Methods for Evidence, Public Health Unit, Sydney Local Health District, Camperdown, NSW 2050, Australia
7 Melbourne School of Population and Global Health, University of Melbourne, Parkville, VIC 3052, Australia
* Correspondence: p.sly@uq.edu.au

Supplementary materials

1 Directed acyclic graph

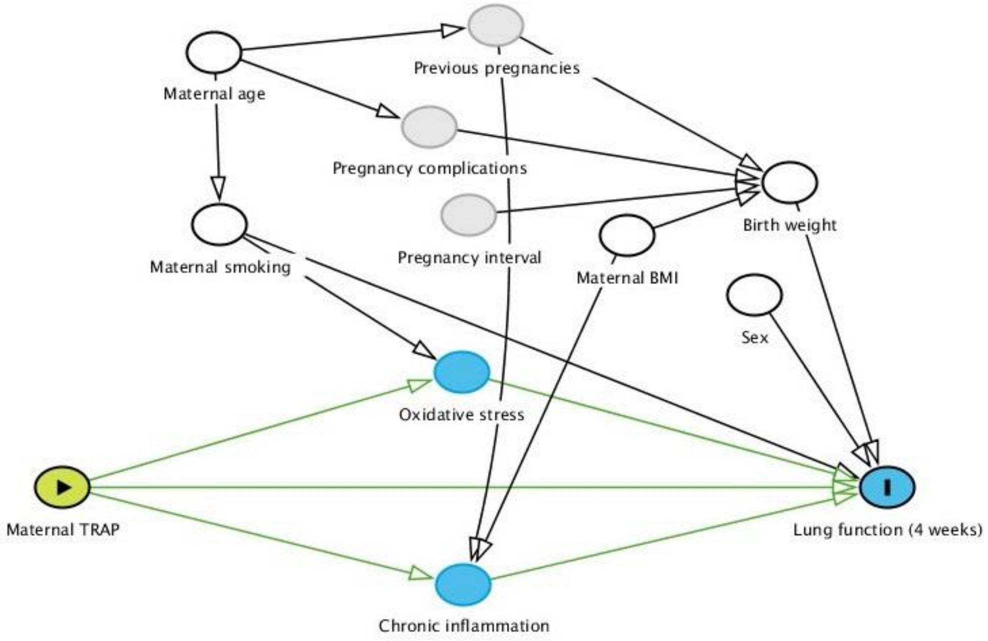

Figure S1: Directed acyclic graph depicted proposed relationships exposure, outcome and covariates. Green shapes and arrows indicate exposure, blue shapes indicate outcome and its antecedents, including potential mediators. White shapes indicate variables which were

included as covariates, while grey shapes are variables proposed to have a relationship on the causal network, but which are unobserved in our models. Maternal TRAP refers to maternal exposure to traffic related air pollution.

2 Summary of exposure, outcome and mediators

Table S1: Summary of exposures and outcomes in the Barwon Infant Study

Variable	mean	sd	median	min	max	IQR
FRC (mL / kg)	87.7	14.9	86.7	52.6	144.0	4.27
LCI (turnovers)	6.79	0.43	6.79	5.410	8.2	0.52
Maternal NO_2 (ppb)	5.58	1.97	5.44	1.983	13.9	2.27
Maternal $PM_{2.5}$ (μg/m^3)	7.50	0.66	7.42	5.932	9.9	0.88
Maternal 8-OHdG (ng/mL) (log)	1.3	0.8	1.3	-1.3	4.1	0.94
Maternal 8-OHGua (ng/mL) (log)	2.9	1.1	3.0	-0.7	6.5	1.09
gPFSox	6.8	0.9	7.00	4.5	9.5	1.5

3 Sensitivity analysis

3.1 SES

Adding socio-economic status to the model (using maternal education level as a proxy measure) of FRC did not significantly change any of the estimates and was not itself significant (β=0.6 mls, 95% CI (-1.7, 2.8), p=0.63).

3.2 Air pollution and maternal smoking interaction (adjusted models)

Table S2: Estimates for lung function at 4 weeks of age (FRC) for maternal exposure to $PM_{2.5}$ and the interaction with maternal smoking in the Barwon Infant Study

	median FRC		
Predictors	*Estimates*	*CI*	*p*
Maternal $PM_{2.5}$ in year before birth (ug/m3)	1.2	-1.5 – 4.0	0.374
$PM_{2.5}$ * maternal smoking	1.6	-5.2 – 8.3	0.650

Models adjusted for sex, birthweight, maternal age, maternal pre-pregnancy BMI and maternal smoking

3.3 Child's passive smoke exposure (adjusted)

Table S3: Estimates for lung function at 4 weeks of age (LCI) for maternal exposure to $PM_{2.5,}$ maternal smoking, and a child's exposure to environmental tobacco smoke at 4 weeks of age in the Barwon Infant Study

	median LCI		
Predictors	*Estimates*	*CI*	*p*
$PM_{2.5}$ (ug/m3)	-0.03	-0.10 – 0.05	0.446
Maternal smoking	-0.04	-0.17 – 0.10	0.612
Child passive smoke exposure	0.01	-0.29 – 0.31	0.947

Models adjusted for sex, birthweight, maternal age, maternal pre-pregnancy BMI and maternal smoking

Table S4: Estimates for lung function at 4 weeks of age (LCI) for maternal exposure to NO_2, maternal smoking, and a child's exposure to environmental tobacco smoke at 4 weeks of age in the Barwon Infant Study

	median LCI		
Predictors	*Estimates*	*CI*	*p*
NO_2 (ppb)	0.01	-0.02 – 0.03	0.580
Maternal smoking	-0.03	-0.17 – 0.10	0.619
Child passive smoke exposure	0.02	-0.27 – 0.32	0.871
Models adjusted for sex, birthweight, maternal age, maternal pre-pregnancy BMI and maternal smoking			

3.4 Child's exposure to $PM_{2.5}$ and lung function (adjusted)

Table S5: Effect estimates for lung function at 4 weeks of age (FRC) for a child's exposure to $PM_{2.5}$ at different timepoints, including an interaction with the oxidative stress genotype, in the Barwon Infant Study.

	median FRC		
Predictors	*Estimates*	*CI*	*p*
$PM_{2.5}$ (mcg/m3) at birth	1.2	-1.3 – 3.6	0.339
$PM_{2.5}$ mcg/m3) at 1 month	0.6	-1.8 – 3.0	0.596
$PM_{2.5}$ mcg/m3) at 1 month * oxidative stress genotype	4.1	-1.9 – 10.0	0.178
Models adjusted for sex, birthweight, maternal age, maternal pre-pregnancy BMI and maternal smoking			

Table S6: Effect estimates for lung function at 4 weeks of age (FRC) for a child's exposure to NO_2 at different timepoints, including an interaction with the oxidative stress genotype, in the Barwon Infant Study.

	median LCI		
Predictors	*Estimates*	*CI*	*p*
$PM_{2.5}$ (mcg/m3) at birth	0.002	-0.069 – 0.072	0.959
$PM_{2.5}$ (mcg/m3) at 1 month	0.012	-0.058 – 0.081	0.742
$PM_{2.5}$ (mcg/m3) at 1 month * oxidative stress genotype	-0.073	-0.246 – 0.099	0.404

Models adjusted for sex, birthweight, maternal age, maternal pre-pregnancy BMI and maternal smoking

3.5 Air pollutants in tertiles

Table S7: Results of linear regression models of maternal exposure to air pollution and infant lung function at 4 weeks of age in the Barwon Infant Study, sensitivity using air pollution variable in tertiles

**Whole cohort**				
		Estimates	*CI*	*p*
**LCI** *n*=275	Medium $PM_{2.5}$	-0.02	-0.14 – 0.10	0.78
	High $PM_{2.5}$	-0.02	-0.14 – 0.10	0.73
	Medium NO_2	-0.09	-0.21 – 0.03	0.13
	High NO_2	-0.01	-0.13 – 0.11	0.91
**FRC** *n*=275	Medium $PM_{2.5}$	1.33	-2.81 – 5.47	0.53
	High $PM_{2.5}$	3.14	-1.04 – 7.32	0.14
	Medium NO_2	2.58	-1.58 – 6.73	0.22
	High NO_2	1.34	-2.85 – 5.53	0.53
**tPTEF/tE** *n*=355	Medium $PM_{2.5}$	-0.01	-0.03 – 0.02	0.47
	High $PM_{2.5}$	0.00	-0.02 – 0.03	0.92
	Medium NO_2	-0.02	-0.05 – 0.00	0.10
	High NO_2	0.001	-0.03 – 0.03	0.97

**Children at high risk of oxidative stress**				
		Estimates	*CI*	*p*
**LCI** *n*=48	Medium $PM_{2.5}$	0.14	-0.17 – 0.46	0.36
	High $PM_{2.5}$	-0.02	-0.34 – 0.30	0.91
	Medium NO_2	0.22	-0.11 – 0.54	0.19
	High NO_2*	0.46	0.10 – 0.82	**0.01**
**FRC** *n*=48	Medium $PM_{2.5}$	0.6	-11.0 – 12.1	0.92
	High $PM_{2.5}$	-0.6	-12.2 – 11.1	0.92
	Medium NO_2	-0.9	-12.6 – 10.8	0.88
	High NO_2^	-13.1	-26.1 – -0.1	**0.05**
**tPTEF/tE** *n*=65	Medium $PM_{2.5}$	-0.04	-0.10 – 0.03	0.31
	High $PM_{2.5}$	-0.02	-0.09 – 0.05	0.64
	Medium NO_2	-0.03	-0.10 – 0.03	0.33
	High NO_2	0.02	-0.05 – 0.09	0.51

Models adjusted for sex, birthweight, maternal age, maternal pre-pregnancy BMI and maternal smoking

* Estimates significantly different to model of low risk OS kids p=0.007

^ Estimates significantly different to model of low risk OS kids p=0.02
